# Acoustic speech markers for schizophrenia-spectrum disorders: a diagnostic and symptom-recognition tool

**DOI:** 10.1017/S0033291721002804

**Published:** 2023-03

**Authors:** J. N. de Boer, A. E. Voppel, S. G. Brederoo, H. G. Schnack, K. P. Truong, F. N. K. Wijnen, I. E. C. Sommer

**Affiliations:** 1 Department of Biomedical Sciences of Cells and Systems and Department of Psychiatry, University of Groningen, University Medical Center Groningen, Groningen, the Netherlands; 2 Department of Psychiatry, University Medical Center Utrecht, Utrecht University & University Medical Center Utrecht Brain Center, Utrecht, the Netherlands; 3 Utrecht Institute of Linguistics OTS, Utrecht University, Utrecht, the Netherlands; 4 Department of Human Media Interaction, University of Twente, Enschede, the Netherlands

**Keywords:** Acoustic, biomarker, language, psychosis, speech

## Abstract

**Background:**

Clinicians routinely use impressions of speech as an element of mental status examination. In schizophrenia-spectrum disorders, descriptions of speech are used to assess the severity of psychotic symptoms. In the current study, we assessed the diagnostic value of acoustic speech parameters in schizophrenia-spectrum disorders, as well as its value in recognizing positive and negative symptoms.

**Methods:**

Speech was obtained from 142 patients with a schizophrenia-spectrum disorder and 142 matched controls during a semi-structured interview on neutral topics. Patients were categorized as having predominantly positive or negative symptoms using the Positive and Negative Syndrome Scale (PANSS). Acoustic parameters were extracted with OpenSMILE, employing the extended Geneva Acoustic Minimalistic Parameter Set, which includes standardized analyses of pitch (F0), speech quality and pauses. Speech parameters were fed into a random forest algorithm with leave-ten-out cross-validation to assess their value for a schizophrenia-spectrum diagnosis, and PANSS subtype recognition.

**Results:**

The machine-learning speech classifier attained an accuracy of 86.2% in classifying patients with a schizophrenia-spectrum disorder and controls on speech parameters alone. Patients with predominantly positive *v.* negative symptoms could be classified with an accuracy of 74.2%.

**Conclusions:**

Our results show that automatically extracted speech parameters can be used to accurately classify patients with a schizophrenia-spectrum disorder and healthy controls, as well as differentiate between patients with predominantly positive *v.* negatives symptoms. Thus, the field of speech technology has provided a standardized, powerful tool that has high potential for clinical applications in diagnosis and differentiation, given its ease of comparison and replication across samples.

## Introduction

In clinical practice, impressions of speech are routinely employed as an element of mental status examination and are a primary source of information in the diagnostic process. Especially in schizophrenia-spectrum disorders, descriptions of speech are used to assess specific symptoms such as alogia (e.g. poverty of speech) and blunted affect (e.g. diminished vocal intonation), as well as positive symptoms such as excitement (e.g. pressured speech; Alpert, Shaw, Pouget, & Lim, 2002). Although there is no consensus on the best sub-categorization of the different symptoms of schizophrenia-spectrum disorders yet, an important distinction is that between positive and negative symptoms. Being able to determine at different moments in time whether a schizophrenia-spectrum patient experiences predominantly positive or negative symptoms is of great clinical importance because these symptom subtypes require different treatment (Fusar-Poli et al., 2015). Moreover, persistent negative symptoms are strongly related to functional outcomes (Lin et al., 2013; Milev, Ho, Arndt, & Andreasen, 2005), further highlighting the need for a reliable distinction between subtypes. From a scientific perspective, reliable identification of relevant subgroups of patients is highly important because the neurobiological underpinnings of their disorders are likely different (Cuthbert & Insel, 2013; Insel, 2014). Consider a patient with grandiose delusions, hallucinations, pressured speech and derailment, *v.* a patient who presents with social withdrawal, alogia and catatonia: although both patients could be classified as having schizophrenia, the processes underlying these clinically non-overlapping symptom-collections might be entirely different.

Traditionally, clinical rating scales, such as the Positive and Negative Syndrome Scale (PANSS; Kay, Fiszbein, & Opfer, 1987) are used to assess both positive and negative symptoms in schizophrenia. However, previous research has stressed the dramatic disparity between speech-based objective measurements of individual characteristics (e.g. flat affect) and scale-based subjective assessments assessed by clinicians; symptom rating scales are, at best, only modestly related to objective measurements of the behavior they intend to reflect (Cohen, Mitchell, & Elvevåg, 2014). Moreover, some PANSS items are highly influenced by experience and preconceptions of the rater, which results in low inter-rater reliability (Kølbæk et al., 2018). In daily practice, these subjective assessments are not performed scale-based, but ad hoc, leading to even higher inter-observer variation. Especially, the assessment of negative symptoms remains problematic, since consensus-based instruments diverge on what should be considered true negative symptoms and what should be considered general/other illness components; such as, for example, cognition (Marder & Galderisi, 2017). Developing objective measurements to complement clinical ratings is thus fundamental to overcome these serious validity concerns with clinical assessments of positive and negative symptoms, and enable development towards measurement-based care (Insel, 2017).

Recent advances in natural language processing have paved the way towards automated speech analyses as a biomarker for psychosis (Corcoran et al., 2020; de Boer et al., 2018; de Boer, Brederoo, Voppel, & Sommer, 2020; Hitczenko, Mittal, & Goldrick, 2020; Tan & Rossell, 2020). Most studies in this field analyze language content, including methods of measuring discourse coherence, syntactic complexity and referential coherence (Bedi et al., 2015; Corcoran et al., 2018; Holmlund et al., 2020; Mota, Furtado, Maia, Copelli, & Ribeiro, 2014; Palaniyappan et al., 2019; Rezaii, Walker, & Wolff, 2019). A source of information that is less commonly used is the acoustic aspects of speech. Articulation and other components involved in speech production can be measured in the acoustic signal, for example by decomposing sound waves into formants; i.e. acoustic resonance frequencies that indicate the position and movement of the articulatory organs when speaking. F1 (first formant) indicates jaw/mouth opening and tongue height, while F2 corresponds to tongue positioning (front/back) and lip rounding. Furthermore, F0, or fundamental frequency, is an acoustic parameter of pitch. Such acoustic speech features (F0 and F2 variability) have been associated with specific negative symptoms (Bernardini et al., 2016; Covington et al., 2012) and have also been utilized as differentiators between individuals with schizophrenia-spectrum disorders and healthy individuals in small samples (Martínez-Sánchez et al., 2015; Tahir et al., 2019), attaining overall classification accuracies varying from 81% to 94%. Although these studies are promising, the larger literature also reports studies that failed to find a difference in acoustic features between schizophrenia-spectrum patients and healthy individuals (Cohen, Mitchell, Docherty, & Horan, 2016; Meaux, Mitchell, & Cohen, 2018).

These inconsistent findings may be due to the fact that several studies took different approaches to computing and standardizing acoustic parameters (Eyben et al., 2016). Individual research groups develop and employ their own set of features, and the studies reported above used acoustic feature sets that overlap only partially. Furthermore, studies often include non-acoustic features (e.g. the usage of determiners in speech) in their analyses. Therefore, the results reported in the literature cannot always be meaningfully compared (de Boer et al., 2020). Indeed, a recent meta-analysis found the existing literature to be highly diverse and unsystematic (Parola, Simonsen, Bliksted, & Fusaroli, 2020). Furthermore, the rapid developments in natural language processing have led to a proliferation in features, often amounting in up to tens of thousands of acoustic parameters (Marmar et al., 2019). While this abundance in features allows capturing many acoustic characteristics, it comes at the cost of overfitting and preventing clear interpretations of the underlying mechanisms (de Boer et al., 2020). We analyzed the speech of patients with a schizophrenia-spectrum disorder and healthy controls using the extended Geneva Acoustic Minimalistic Parameter Set (eGeMAPS) for acoustic analyses (Eyben et al., 2016). This parameter set was developed to provide a baseline for affective speech processing, in order to ensure easy replication and to improve comparability of parameters.

Overall, some recent studies have shown promising results in identifying schizophrenia-spectrum disorders from healthy controls utilizing automated analysis of acoustic speech patterns (Martínez-Sánchez et al., 2015; Tahir et al., 2019). Yet, inconsistencies remain and replications in large samples using interpretable and standardized speech parameters are required (Meaux et al., 2018; Parola et al., 2020). Thus, the first aim of the current study is to replicate the diagnostic potential of standardized speech parameters in a large sample of patients with a schizophrenia-spectrum disorder. The second aim is to explore the value of acoustic analyses in differentiating between patients who at that time experience predominantly positive *v.* negative psychotic symptoms. Third, we aim to explore the relation between acoustic features and cognitive and psychotic symptoms, as well as the relation with antipsychotic medication. Based on previous study from our group, we specifically expect increased pauses to be related to the use of typical antipsychotics (de Boer, Voppel, Brederoo, Wijnen, & Sommer, 2020).

## Methods

### Participants

Data from 142 Dutch patients with a schizophrenia-spectrum disorder and 142 healthy age- and sex-matched controls from the same community were collected between 2015 and 2020. All procedures were approved by the Ethical Committee of the University Medical Center Utrecht. Psychiatric diagnoses were confirmed by the Structured Clinical Interview for DSM-IV (SCID; First, 2014), the Comprehensive Assessment of Symptoms and History (CASH; Andreasen, Flaum, & Arndt, 1992) or the Mini-International Interview (MINI; Sheehan et al., 1998), depending on the study the participants originally enrolled in. Healthy controls were screened for the absence of previous or current mental illness. Symptom severity and cognitive functioning were rated by consensus rating of two trained researchers, blinded to phonetic analysis, with the PANSS (Kay et al., 1987) and Brief Assessment of Cognition (BACS; Keefe et al., 2004). The patients were categorized as having predominant negative *v.* positive symptoms based on PANSS scores. Since there is no consensus in the literature on cutoff scores for such subcategorizations, a median split was used. Patients were therefore categorized as having predominantly negative symptoms if they had a greater score on the negative than on the positive subscale of the PANSS, and a negative subscale score ⩾ the median, being 12. The patients with predominantly positive symptoms consisted of patients with PANSS positive subscale scores that were greater than their negative subscale scores, and a positive subscale score ⩾ the median, being 9. Of the 142 schizophrenia-spectrum cases, 44 had predominantly positive symptoms and 45 had predominantly negative symptoms, the remaining patients had an equal combination of positive and negative symptoms.

Patients were divided into two categories based on different dopamine binding profiles, namely patients with (1) low dopamine D2 receptor (D2R) occupancy drugs (i.e. quetiapine, paliperidone, olanzapine and clozapine) or (2) high D2R occupancy drugs (i.e. aripiprazole, risperidone, flupentixol, amisulpride and haloperidol), following a previous report by our group (de Boer et al., 2020). Antipsychotic drug dosages were recalculated into chlorpromazine equivalents to evaluate the effect of dosage between the drugs (Leucht et al., 2014).

## Procedure

### Speech recording

Semi-structured interviews varying from 5 to 30 min in length (average 11 min) were conducted using a set of neutral open-ended questions. For an elaborate description of this methodology, see previous reports by our group (de Boer, van Hoogdalem, et al., 2020; de Boer, Voppel, et al., 2020; Voppel, de Boer, Brederoo, Schnack, & Sommer, 2021). AKG-C544l cardioid microphones were used to record the participant's and interviewer's speech onto two different channels. Speech was digitally recorded onto a Tascam DR40 solid state recorder, at a sampling rating of 44.1 kHz with 16-bit quantization. Head-worn microphones were used to keep the distance between the mouth and the microphone as constant as possible (2 cm), since this has a considerable effect on recorded vocal loudness. Microphones were placed at a 45° angle from the participant's mouth in order to prevent aerodynamic noise.

### Speech pre-processing

To remove crosstalk (i.e. speech from the interviewer on the participant's audio channel), the following steps were taken: (1) the ‘annotate to text grid silences’ function in PRAAT (Boersma & Weenink, 2013) was used on the interviewer's channel (settings: minimum pitch 100 Hz, time step 0.0, silence threshold −30.0 dB, minimum silence duration 1.0 s, minimum sounding duration 0.1 s); (2) all resulting speech segments in which the interviewer was silent were selected on the participants channel and (3) the resulting speech segments were concatenated into a new audio file containing only segments of speech of the participant.

### Acoustic parameters

The eGeMAPS parameter set was extracted from the participant's speech using OpenSMILE (Eyben, Weninger, Gross, & Schuller, 2013). eGeMAPS provides arithmetic means and coefficients of variation [standard deviation (s.d.) normalized by the arithmetic mean] for each parameter. A total of 88 parameters were computed at the speaker level and were used for feature selection and classification model building. For a full overview of the parameters, see online Supplementary Table S1. The parameters can be divided into the following types (Eyben et al., 2013): temporal (e.g. speech rate; six parameters), frequency (e.g. fundamental frequency; 24 parameters), spectral [e.g. Mel-frequency cepstral coefficients (MFCCs), relative energy in different frequency bands; 43 parameters] and energy/amplitude (e.g. intensity; 14 parameters).

### Cognitive functioning

Cognition was assessed in all patients using the BACS (Keefe et al., 2004) which consists of the following tasks: (1) List learning – Verbal memory; (2) Digit sequencing - Working memory; (3) Token motor task - Motor speed; (4) Category Instances and Controlled Oral Word Association Test - Verbal fluency; (5) Symbol coding - Attention and information processing speed and (6) Tower of London - Executive function. Individual BACS scores were converted into standardized *Z*-scores that are corrected for age and gender based on previously published norm scores (Keefe et al., 2008).

### Statistical analysis

For categorical variables, a χ^2^ test, and for continuous variables analysis of variance (ANOVAs) were used to assess differences between groups in demographic characteristics. Following previous research (Marmar et al., 2019), random forest algorithms were used to build machine-learning classifiers using acoustic speech parameters to differentiate between schizophrenia-spectrum patients and healthy controls, and to classify symptom patterns (i.e. predominantly positive *v.* predominantly negative symptoms). In this study, random forest classifiers were built using the 88 speech markers, using leave-ten-out cross-validation to divide the data into test and train sets. Random forests are based on multiple classification trees. The nodes in these decision trees are based on a binary ‘split’ of a predictor, aimed at minimizing misclassification in a training subset of the data. The process continues recursively until a tree is formed that does not improve from further splits or nodes. The probability of being a member of a certain target class, is the fraction of that class in the terminal node into which they fall. Once trees are iteratively built, per-case classification is performed on the testing subset. The resulting probability estimates are used to generate a receiver operator curve (ROC) and its area under the curve (AUC). From the trained trees, the importance of a specific predictor (Gini-importance score) is estimated by setting the predictor to a random number, and comparing the difference in predictive power (AUCs) to the final model, measuring how much worse the model becomes when replacing the predictor with random data. Gini-importance scores are average scores over all recursive trees, and can only be interpreted in relation to each other. Bivariate Pearson correlations were performed to analyze associations between acoustic variables and clinical characteristics in the patients for the top 10 features in both models. Correlation analyses were corrected for multiple comparison using false-discovery rate (FDR). Alpha was set to 0.05 for all analyses.

## Results

The schizophrenia-spectrum patients and controls did not differ significantly in age, sex or parental educational levels (see Table 1). The patients overall received less education than the controls, which is to be expected given that the first psychosis often occurs during educational years. The schizophrenia-spectrum patients with predominantly positive symptoms had a longer illness duration than the patients with predominantly negative symptoms. These subgroups did not differ significantly on age, sex and parental educational levels. The patients with predominantly positive symptoms had a longer illness duration than the patients with predominantly negative symptoms (Table 1).

**Table 1.** Demographics

	HC	SSD			SSD *v*. HC		Predominantly positive *v*. negative symptoms	
		Full sample	Predominantly positive symptoms	Predominantly negative symptoms	Statistic	*p* value	Statistic	*p* value
Sample size, *n*	142	142	44	45	–	–	–	–
Male sex, *n* (%)	86 (60.6)	101 (71.1)	36 (81.8)	32 (71.1)	χ^2^ = 3.52	0.061	χ^2^ = 1.42	0.234
Age, mean years (s.d.)	34.5 (13.75)	32.4 (12.53)	34.5 (13.03)	26.7 (9.95)	*F* = 1.65	0.200	*F* = 3.84	0.053
Years of education, mean (s.d.)	15.0 (1.77)	13.2 (2.38)	12.9 (2.63)	13.4 (13.44)	*F* = 51.4	<0.001**	*F* = .987	0.323
Years of education parents, mean (s.d.)	12.8 (2.85)	12.4 (2.89)	12.3 (2.89)	12.8 (2.67)	*F* = 1.13	0.288	*F* = .671	0.415
Duration disease years, mean (s.d.)	–	6.4 (10.26)	9.5 (12.35)	3.2 (6.76)			*F* = 9.00	0.004**
Chlorpromazine equivalent, mean (s.d.)	–	237.0 (154.53)	268.5 (173.67)	228.7 (136.58)			*F* = 1.31	0.257
DSM diagnosis, *n* (%)							χ^2^ = 3.89	0.506
Schizophrenia	–	55 (38.7)	19 (43.2)	21 (46.7)				
Schizophreniform disorder	–	7 (4.9)	2 (4.5)	2 (4.4)				
Schizoaffective disorder	–	15 (10.6)	6 (13.6)	2 (4.4)				
Psychosis NOS	–	65 (45.8)	17 (38.6)	20 (44.4)				
PANSS, mean (s.d.)								
Total	–	47.0 (15.30)	51.3 (12.36)	55.2 (9.61)			*F* = 2.65	0.107
Positive	–	10.5 (4.70)	14.7 (4.45)	10.2 (2.80)			*F* = 32.5	<0.001**
Negative	–	12.3 (5.35)	10.7 (3.36)	17.6 (3.88)			*F* = 80.3	<0.001**
General	–	24.2 (7.94)	26.0 (6.53)	27.4 (6.21)			*F* = 1.07	0.303

HC: healthy controls; SSD: schizophrenia-spectrum disorder patients.

Psychotic symptom subgroup analyses were performed in those patients that met criteria for either predominantly positive or negative symptoms. These subgroups are a subset (*n* = 89) of the full schizophrenia-spectrum disorder sample (*n* = 142).

** Indicates *p* value <0.01.

### Diagnostic classifier

The trained 10-fold cross-validated classifier had an accuracy of 86.2% with an AUC of 0.92 in distinguishing schizophrenia-spectrum patients from healthy controls. Sensitivity was 85.1% and specificity 87.2% (see Table 2).

**Table 2.** Classification of performance metrics

	Sens (95% CI)	Spec (95% CI)	NPV	PPV	Accuracy	AUC (95% CI)
Diagnostic classifier (SSD *v*. HC)	0.851 (0.78-0.90)	0.872 (0.81-0.92)	0.854 (0.79-0.90)	0.870 (0.80-0.92)	0.8616	0.920 (0.89-0.95)
Symptom classifier (positive *v*. negative symptoms)	0.659 (0.51-0.78)	0.800 (0.66-0.89)	0.706 (0.57-0.81)	0.763 (0.61-0.87)	0.7416	0.760 (0.66-0.86)

Legend: SSD: schizophrenia-spectrum disorder patients, HC: healthy controls, sens: sensitivity, spec: specificity, CI: confidence interval, NPV: negative predictive value, PPV: positive predictive value, AUC: area under the curve.

Table 3 lists the ten acoustic parameters with the highest importance scores in the final model, as well as the means and s.d.s of these parameters in the schizophrenia-spectrum patients and controls. For a full list of Gini-importance scores, see online Supplementary Table S1. Three of the top parameters in the model were temporal (voiced segment rate, unvoiced segment length and s.d.), indicating short, fragmented speech segments and longer pauses in the schizophrenia-spectrum patients compared to controls. Patients with a schizophrenia-spectrum disorder were further classified using spectral parameters between groups, namely a reduced mean spectral slope of voiced and unvoiced regions (indicating a more tensed voice in the patients) and reduced spectral flux variation (i.e. less difference between spectra measured between two consecutive time windows, which may indicate more monotone speech). Patients were further characterized by reduced variation of F2 bandwidth (i.e. vowel bandwidth, can indicate breathiness). Moreover, the energy of the first and third formant as compared to pitch (F0) differed from healthy controls, indicating differences in intonation characteristics and voice quality. The patients showed a larger variation in loudness compared to the controls.

**Table 3.** Top ten acoustic parameters in the diagnostic classifier (schizophrenia-spectrum disorder *v*. healthy control)

Ranking	Parameter	Description	May reflect	Feature type	Mean HC (s.d.)	Mean SZ (s.d.)	*F*-statistic	*p* value
1	Voiced segments per second (mean)	Average number of continuous voiced regions per second (more segments indicates more fragmented speech with short speech segments).	Fragmentedness of speech	Temporal	1.25 (0.014)	1.75 (0.559)	103.3	<0.001**
2	Slope UV 500–1500 (mean)	Mean spectral Slope 0–500 Hz and 500–1500 Hz of unvoiced regions; linear regression slope of the logarithmic power spectrum within the two given bands.	Tension	Spectral	−0.009 (0.0024)	−0.006 (0.0025)	75.1	<0.001**
3	F2 bandwidth (s.d.)	s.d. of the bandwidth of second formant	Breathiness	Frequency	0.36 (0.004)	0.33 (0.047)	20.0	<0.001**
4	Unvoiced segment length (mean)	The mean length of unvoiced regions (F0 = 0; approximating pauses).	Pause length	Temporal	0.20 (0.006)	0.26 (0.108)	33.9	<0.001**
5	Spectral flux (s.d.)	s.d. of spectral flux difference of the spectra of two consecutive frames.	Monotone speech	Spectral	1.26 (0.178)	1.38 (0.241)	20.3	<0.001**
6	Loudness (s.d.)	Loudness; estimate of perceived signal intensity from an auditory spectrum.	Loudness	Energy/amplitude	0.96 (0.119)	1.06 (0.161)	33.7	<0.001**
7	Slope V 500–1500 (mean)	Mean spectral Slope 0–500 Hz and 500–1500 Hz of voiced regions; linear regression slope of the logarithmic power spectrum within the two given bands.	Tension	Spectral	−0.02 (0.003)	−0.02 (0.003)	26.8	<0.001**
8	F3 log relative amplitude (s.d.)	s.d. of relative energy of the third formant's center frequency to the energy of the spectral peak at F0.	Vowel pronunciation	Spectral	−0.73 (0.071)	−0.68 (0.093)	27.2	<0.001**
9	F1 log relative amplitude (mean)	Mean relative energy of the first formant's center frequency to the energy of the spectral peak at F0.	Vowel pronunciation	Spectral	−96.3 (14.29)	−105.7 (17.96)	23.6	<0.001**
10	Unvoiced segment length (s.d.)	s.d. of unvoiced regions (F0 = 0; approximating pauses).	Pause length	Temporal	0.39 (0.140)	0.50 (0.243)	75.1	<0.001**

s.d., standard deviation; UV, unvoiced; V,  voiced.

Ranking based on average Gini-importance scores.

** indicates *p* value <0.01.

### Psychotic symptoms classifier

The trained 10-fold cross-validated model had an accuracy of 74.2% with an AUC–ROC of 0.76 in distinguishing patients with predominantly negative from those with predominantly positive symptoms. Sensitivity was 65.9% and specificity 80.0% (see Table 2). Table 4 lists the top ten acoustic parameters in the trained model, as well as the means and s.d.s of these parameters in the predominant positive and negative symptom groups. For a full list of Gini-importance scores, see online Supplementary Table S2. Patients with positive symptoms had a smaller spectral slope indicating a smaller difference in energy between low and high frequencies. Other spectral parameters did not differ between groups. Seven of the top ten parameters were frequency parameters (i.e. relating to the way vowels are pronounced); patients with positive symptoms had a smaller F1 and F2 bandwidth (i.e. vowel bandwidth, indicating breathiness), lower mean F1 frequencies, less jitter variation (i.e. roughness or voice quality) and less variation in F3 frequency (i.e. vowel frequency).

**Table 4.** Top ten acoustic parameters in the psychotic symptoms classifier (positive *v*. negative symptoms)

Ranking	Parameter	Description	May reflect	Feature type	Mean predominantly positive patients (s.d.)	Mean predominantly negative patients (s.d.)	*F*-statistic	*p* value
1	Slope UV 500–1500 (mean)	Mean spectral slope 0–500 Hz and 500–1500 Hz of unvoiced regions; linear regression slope of the logarithmic power spectrum within the two given bands.	Tension	Spectral	−0.03 (0.012)	−0.04 (0.017)	10.6	0.002**
2	MFCC3 (s.d.)	s.d. of Mel-Frequency Cepstral Coefficient 3.	Timbre	Spectral	0.70 (0.149)	0.68 (0.218)	0.824	0.366
3	F1 bandwidth (mean)	Mean bandwidth of first formant.	Breathiness	Frequency	1283.7 (54.01)	1318.0 (43.78)	10.9	0.001**
4	F3 frequency (s.d.)	s.d. of third formant frequency.	Vowel pronunciation	Frequency	0.11 (0.017)	0.12 (0.012)	4.15	0.045*
5	Jitter (s.d.)	s.d. of jitter; deviations in individual consecutive F0 period lengths.	Voice quality	Frequency	1.77 (0.240)	1.88 (0.255)	4.7	0.034*
6	F2 bandwidth (mean)	Mean bandwidth of second formant.	Breathiness	Frequency	995.9 (76.23)	1034.9 (58.34)	7.37	0.008**
7	F1 frequency (mean)	Mean frequency of first formant.	Vowel pronunciation	Frequency	511.0 (58.91)	521.3 (41.84)	0.949	0.333
8	Alpha ratio voiced (s.d.)	s.d. of the alpha ratio of voiced segments; ratio of the summed energy from 50 Hz to 1000 Hz and 1–5 kHz	Voice quality	Spectral	−0.36 (0.031)	−0.35 (0.043)	1.45	0.232
9	F2 frequency (s.d.)	s.d. of second formant frequency.	Vowel pronunciation	Frequency	1507.5 (78.60)	1528.4 (68.55)	2.97	0.089
10	F3 bandwidth (mean)	Mean bandwidth of third formant.	Breathiness	Frequency	866.0 (84.45)	907.4 (66.85)	6.59	0.012*

Ranking based on average Gini-importance scores. s.d.: standard deviation; UV:  unvoiced; V:  voiced. ** indicates *p* value <0.01, * indicates *p* value <0.05.

### Associations between acoustic variables and clinical characteristics

#### Psychotic and cognitive symptoms

Pearson correlations for the top ten parameters from both classifiers with PANSS positive, negative and general and composite BACS scores are presented in Table 5. Acoustic variables correlated most strongly with negative and to a lesser degree general PANSS scores. After FDR correction, no significant associations with cognitive functioning were found.

**Table 5.** Associations between top acoustic parameters and psychotic and cognitive symptoms

Classifier and ranking	Acoustic parameters	PANSS Positive	PANSS Negative	PANSS General	BACS composite
Diagnostic 1	Voiced segments per second (mean)	−0.299**	0.005	−0.170*	−0.113
Diagnostic 2	Slope UV 500–1500 (mean)	**−0**.003	−0.087	0.031	0.063
Diagnostic 3	F2 bandwidth (s.d.)	**−0**.087	−0.160	−0.113	−0.041
Diagnostic 4	Unvoiced segment length (mean)	0.096	0.163	0.143	−0.017
Diagnostic 5	Spectral flux (s.d.)	0.087	0.250**	0.187*	−0.058
Diagnostic 6	Loudness (s.d.)	0.050	0.264**	0.193*	−0.093
Diagnostic 7	Slope V 500–1500 (mean)	0.096	0.109	0.077	−0.007
Diagnostic 8	F3 log relative amplitude (s.d.)	0.111	0.351**	0.271**	−0.132
Diagnostic 9	F1 log relative amplitude (mean)	**−0**.095	−0.298**	−0.226**	0.125
Diagnostic 10	Unvoiced segment length (s.d.)	0.106	0.094	0.105	0.017
Symptoms 1	Slope UV 500–1500 (mean)	**−0**.003	−0.087	0.031	0.063
Symptoms 2	MFCC3 (s.d.)	**−0**.004	−0.114	−0.067	0.006
Symptoms 3	F1 bandwidth (mean)	**−0**.063	0.261**	0.180*	−0.074
Symptoms 4	F3 frequency (s.d.)	0.010	0.201*	0.153	0.033
Symptoms 5	Jitter (s.d.)	**−0**.098	0.047	−0.018	−0.115
Symptoms 6	F2 bandwidth (mean)	0.012	0.284**	0.205*	−0.017
Symptoms 7	F1 frequency (mean)	**−0**.102	0.021	0.007	−0.180*
Symptoms 8	Alpha ratio voiced (s.d.)	0.079	0.248**	0.297**	−0.107
Symptoms 9	F2 frequency (s.d.)	**−0**.042	0.097	0.140	0.004
Symptoms 10	F3 bandwidth (mean)	0.054	0.271**	0.171*	0.024

Legend: Reported values are Pearson's *r* values. ** indicates *p* value <0.01, * indicates *p* value <0.05. Bold values remained significant after FDR correction for multiple comparisons.

#### Antipsychotic medication

Pearson correlations between chlorpromazine equivalents and all 88 acoustic parameters revealed only one weak association; slope V500–1500 (s.d.) was correlated with chlorpromazine equivalent dose (*r* = −0.207, *p* = 0.019). This association did not remain significant after FDR correction. A multivariate analysis of variance (MANOVA) analysis comparing patients with low DR2 occupancy drugs with patients using high DR2 occupancy drugs, and chlorpromazine equivalent dose as a covariate, revealed no significant overall group effect of medication type (*F*_(1, 85)_ = 1.191, *p* = 0.269) or dose (*F*_(1, 85)_ = 1.260, *p* = 0.206) on the acoustic parameters. An ANOVA analysis comparing the two medication groups on pause duration (mean unvoiced segment length) specifically, with chlorpromazine equivalent dose as a covariate, revealed a significant main effect of drug type (*F*_(1, 126)_ = 4.532, *p* = 0.035, partial *η*^2^ = 0.035). Antipsychotic dose showed no significant effect on pause duration (*p* = 0.642). Patients with high DR2 occupancy drugs showed a greater pause duration (mean = 0.28) than the patients with low DR2 occupancy drugs (mean = 0.24).

## Discussion

This study confirms previous research in showing that patients with a schizophrenia-spectrum disorder can be reliably distinguished from healthy control participants by using automated speech-based techniques, in a large sample. We extend the current literature by further showing that patients with a schizophrenia-spectrum disorder can be divided into those with predominantly positive *v.* negative symptoms based on their speech acoustics.

By using standardized acoustic measures, high sensitivity, specificity and accuracy could be achieved in differentiating patients from controls. The model assigns higher probability to a schizophrenia-spectrum diagnosis when speech is fragmented (i.e. frequent short-speech segments), has a decreased spectral slope (i.e. less difference in energy between high- and low-speech frequencies) and longer pauses. We further demonstrated that speech parameters can be used to identify subjects with positive and negative symptoms in schizophrenia-spectrum disorders. This model assigns higher probability to positive symptoms when there is less variation in jitter (i.e. roughness of the voice), as well as differences in vowel quality (e.g. breathiness and rounding of vowels, indicated by reduced variation in F3 formant frequencies, a lower F1 formant frequency and a smaller F1 and F2 formant bandwidth (i.e. reach of the formants, affected by jaw/mouth opening as well as tongue and lip positioning). This indicates that speech-markers do not only differentiate between ‘normal’ and ‘disturbed’ speech, but also (reliably) reflect positive and negative symptoms.

Our results showed that temporal (i.e. timing related) parameters are highly important in classifying patients and controls, as three out of six temporal parameters were included in the top ten parameters. This is an interesting finding since temporal parameters comprise only 6.8% of the acoustic parameters used. These findings are in correspondence with a recent meta-analysis indicating that temporal aspects such as speech rate and pausing patterns are often disturbed in schizophrenia-spectrum disorders (Cohen et al., 2014; Parola et al., 2020). Contrastingly, temporal parameters were not among the top ten parameters characterizing positive *v.* negative psychotic symptoms. Timing-related parameters are not specific to a single-mental process. In this study, we did not find an association between overall cognitive functioning and the timing related parameters in our models. However, a more detailed exploration of this relation could reveal associations between acoustic-timing and specific aspects of cognitive functioning. Reduced speech rate can, for example, be the result of slower word-retrieval, slower processing speed, slower articulation or increased distractibility. Recent work has shown that increased pausing in schizophrenia-spectrum disorders is mostly observed clause-initial, suggesting that these patients require more time to formulate a sentence (Çokal et al., 2019). Moreover, in a previous report by our group we found that patients with increased pausing specifically had difficulties with memory-related tasks (Oomen et al., 2021). We found that several of the acoustic parameters in our models were associated with negative and to a lesser degree general PANSS scores, including loudness, formant bandwidth and amplitude. These findings suggest that patients with high negative PANSS scores have a more breathy voice and reduced voice quality. Moreover, voiced segments per s was negatively associated with positive PANSS scores, suggesting that patients with high positive PANSS have less fragmented speech. A thorough exploration of these associations is beyond the scope of the current study; further research is needed to explore how acoustic aspects are related to individual PANSS items.

Our results revealed no effect of antipsychotic medication dose on the acoustic parameters, which is in accordance with our previous work (de Boer et al., 2020). Furthermore, although we did not find an overall effect of drug type on the acoustic variables, we replicated our previous work in showing that the use of high DR2 drugs was associated with increased pausing (de Boer et al., 2020).

Research on spectral profile parameters suggests they are mostly associated with vocal valence, such as angry speech, as well as the control over emotions (Eyben et al., 2016; Goudbeek & Scherer, 2010). Spectral slope and pitch variation have also been associated with emotional stress (Simantiraki, Giannakakis, Pampouchidou, & Tsiknakis, 2016). Frequency related parameters have been associated with arousal, alertness and engagement in the conversation (Goudbeek & Scherer, 2010). Applying these interpretations to our findings suggests that patients with positive *v.* negative symptoms show differences in terms of arousal, alertness and engagement (frequency related parameters), and to a lesser degree in vocal valence (e.g. angry/happy). However, it should be noted that there is little research on the interpretation of acoustic parameters in relation to psychopathology. Most research has been conducted in healthy individuals. Interpreting the different parameters in relation to schizophrenia-spectrum disorders or psychotic symptoms therefore remains speculative for now.

Of note, there is some circularity in this line of research. On the one hand PANSS scores are – for some part – based on a clinical interpretation of speech (e.g. pauses; Alpert, Pouget, & Silva, 1995), on the other hand acoustic speech measurements are validated by their association with negative symptoms. Clinicians describe a persons' speech as being ‘flat’, ‘monotonous’ or ‘aprosodic’, which is then traced back to smaller F0 range and decreased variability in F2 (Compton et al., 2018). Speech analysis should be used routinely to understand the changes in speech during psychosis, and should be favored over less precise clinical descriptions of spoken language. Phonetic parameters have (relatively) clear underlying biological mechanisms (Eyben et al., 2016), while we do not know what a clinician hears when he describes a patient's speech as ‘monotonous’. For example, there is evidence that clinicians are influenced by speaking patterns in assessing the severity of all negative symptoms; when pause length is manipulated in spoken language, clinicians tend to rate other (non-speech) negative symptoms as more severe (Alpert et al., 1995).

This line of research is still in the proof of concept phase and will face a number of challenges before it can be clinically implemented (Dukart, Weis, Genon, & Eickhoff, 2021). For example, biases in machine learning (Schnack, 2020), privacy issues, generalizability and technical pitfalls should be dealt with in the first place (Jacobson et al., 2020; Starke, De Clercq, Borgwardt, & Elger, 2020). After these critical hurdles are taken, we envision acoustic analysis to have several valuable clinical implementations. For example, speech analyses could be used in primary care facilities as an initial screening instrument. Currently, diagnostic accuracy in primary care is low as the personnel is not specifically trained in psychiatric problems. Individuals with a high likelihood of a schizophrenia-spectrum disorder could for example be prioritized for a referral to a mental health care professional. Moreover, we expect that accuracy of models like these will significantly improve from the combination of different types of analyses, such as a combination of acoustic, semantic and syntactic analyses (de Boer et al., 2020). With sufficient levels of accuracy, possible clinical implementations could include relapse prediction, prediction of treatment efficacy, prognosis, early diagnosis and differential diagnosis.

Although the temporal stability of speech anomalies in schizophrenia-spectrum disorders has scarcely been studied (Cohen et al., 2019), phonetic research has shown that some acoustic properties such as formant trajectories are highly stable over time (Hasan, Jamil, & Rahman, 2004; Ingram, Prandolini, & Ong, 2013; Nolan & Grigoras, 2005), making them suitable even for speaker identification. If acoustic speech properties are indeed highly personal and stable, deviations from such a stable personal pattern may very well be a cue for relapse into psychosis in an individual person. Further longitudinal studies are required to ascertain how speech disturbances in schizophrenia-spectrum disorders develop over time (Arevian et al., 2020; Cohen et al., 2019).

There were a number of limitations in the study. First, although conservative leave-ten-out cross-validation was employed, there remains a risk of overfitting. Replication in an independent dataset is therefore required. Secondly, although several variables that can influence the results have been explored, not all factors could be controlled for. For example, we had no data on smoking behavior, length or weight, which are known to influence some aspects of speech. Moreover, the recordings were made in different rooms, which may have affected some of the acoustic aspects of speech. Therefore, future research should assess the specificity of the classifier by controlling for these factors and by testing its performance in differential diagnosis of psychiatric disorders. Finally, it should be noted that we averaged the acoustic parameters over the interview duration (mean duration 10.8 min), which precludes dynamic differences over time. Further research should therefore also incorporate dynamic aspects of speech acoustics, such as convergence between speech partners, to fully acknowledge the within-subject variety of speech acoustics as well.

In conclusion, these findings show that standardized acoustic speech parameters can be used to accurately classify patients with a schizophrenia-spectrum disorder and healthy controls, as well as differentiate between patients with predominantly positive *v.* negatives symptoms. Our findings support the usefulness of computational tools to characterize complex human behavior such as speech. We strongly encourage the use of standardized open-source software packages such as eGeMAPS since they ensure easy comparison and replication across samples and even cross-linguistically.

## Data Availability

The data that support the findings of this study are available on request from the corresponding author. The data are not publicly available as they contain information that could compromise research participant privacy or consent.

## Supplementary material

For supplementary material accompanying this paper visit https://doi.org/10.1017/S0033291721002804. click here to view supplementary material
