# Supplementary material for: Acoustic speech markers for schizophrenia-spectrum disorders: a diagnostic and symptom-recognition tool
Source: Psychol Med. 2021 Aug 4;53(4):1302–12. doi: 10.1017/S0033291721002804 (PMC10009369; doi:10.1017/S0033291721002804)
Supplement: Supplementary file 1 [file S0033291721002804sup001.docx]

**Supplementary material**

**Supplementary Table 1. Gini-importance scores of the acoustic parameters in the diagnostic classifier (schizophrenia-spectrum disorder vs healthy control)**

| **Parameter** | **Gini-importance** |
| --- | --- |
| F0semitoneFrom27.5Hz_sma3nz_amean_numeric | 1.00 |
| F0semitoneFrom27.5Hz_sma3nz_stddevNorm_numeric | 1.27 |
| F0semitoneFrom27.5Hz_sma3nz_percentile20.0_numeric | 1.40 |
| F0semitoneFrom27.5Hz_sma3nz_percentile50.0_numeric | 0.72 |
| F0semitoneFrom27.5Hz_sma3nz_percentile80.0_numeric | 0.74 |
| F0semitoneFrom27.5Hz_sma3nz_pctlrange02_numeric | 1.01 |
| F0semitoneFrom27.5Hz_sma3nz_meanRisingSlope_numeric | 1.63 |
| F0semitoneFrom27.5Hz_sma3nz_stddevRisingSlope_numeric | 1.77 |
| F0semitoneFrom27.5Hz_sma3nz_meanFallingSlope_numeric | 1.42 |
| F0semitoneFrom27.5Hz_sma3nz_stddevFallingSlope_numeric | 1.33 |
| loudness_sma3_amean_numeric | 0.88 |
| loudness_sma3_stddevNorm_numeric | 2.59 |
| loudness_sma3_percentile20.0_numeric | 1.00 |
| loudness_sma3_percentile50.0_numeric | 1.16 |
| loudness_sma3_percentile80.0_numeric | 1.00 |
| loudness_sma3_pctlrange02_numeric | 1.24 |
| loudness_sma3_meanRisingSlope_numeric | 0.75 |
| loudness_sma3_stddevRisingSlope_numeric | 0.89 |
| loudness_sma3_meanFallingSlope_numeric | 0.75 |
| loudness_sma3_stddevFallingSlope_numeric | 0.65 |
| spectralFlux_sma3_amean_numeric | 0.76 |
| spectralFlux_sma3_stddevNorm_numeric | 3.31 |
| mfcc1_sma3_amean_numeric | 0.82 |
| mfcc1_sma3_stddevNorm_numeric | 1.07 |
| mfcc2_sma3_amean_numeric | 1.12 |
| mfcc2_sma3_stddevNorm_numeric | 0.85 |
| mfcc3_sma3_amean_numeric | 1.14 |
| mfcc3_sma3_stddevNorm_numeric | 1.22 |
| mfcc4_sma3_amean_numeric | 1.19 |
| mfcc4_sma3_stddevNorm_numeric | 1.34 |
| jitterLocal_sma3nz_amean_numeric | 1.52 |
| jitterLocal_sma3nz_stddevNorm_numeric | 1.81 |
| shimmerLocaldB_sma3nz_amean_numeric | 1.14 |
| shimmerLocaldB_sma3nz_stddevNorm_numeric | 1.28 |
| HNRdBACF_sma3nz_amean_numeric | 0.73 |
| HNRdBACF_sma3nz_stddevNorm_numeric | 0.83 |
| logRelF0H1H2_sma3nz_amean_numeric | 1.00 |
| logRelF0H1H2_sma3nz_stddevNorm_numeric | 0.83 |
| logRelF0H1A3_sma3nz_amean_numeric | 0.62 |
| logRelF0H1A3_sma3nz_stddevNorm_numeric | 1.07 |
| F1frequency_sma3nz_amean_numeric | 1.30 |
| F1frequency_sma3nz_stddevNorm_numeric | 1.68 |
| F1bandwidth_sma3nz_amean_numeric | 1.35 |
| F1bandwidth_sma3nz_stddevNorm_numeric | 1.08 |
| F1amplitudeLogRelF0_sma3nz_amean_numeric | 2.16 |
| F1amplitudeLogRelF0_sma3nz_stddevNorm_numeric | 1.93 |
| F2frequency_sma3nz_amean_numeric | 1.11 |
| F2frequency_sma3nz_stddevNorm_numeric | 0.88 |
| F2bandwidth_sma3nz_amean_numeric | 1.12 |
| F2bandwidth_sma3nz_stddevNorm_numeric | 3.89 |
| F2amplitudeLogRelF0_sma3nz_amean_numeric | 1.54 |
| F2amplitudeLogRelF0_sma3nz_stddevNorm_numeric | 1.57 |
| F3frequency_sma3nz_amean_numeric | 0.87 |
| F3frequency_sma3nz_stddevNorm_numeric | 0.89 |
| F3bandwidth_sma3nz_amean_numeric | 1.36 |
| F3bandwidth_sma3nz_stddevNorm_numeric | 1.04 |
| F3amplitudeLogRelF0_sma3nz_amean_numeric | 1.42 |
| F3amplitudeLogRelF0_sma3nz_stddevNorm_numeric | 2.25 |
| alphaRatioV_sma3nz_amean_numeric | 0.75 |
| alphaRatioV_sma3nz_stddevNorm_numeric | 1.02 |
| hammarbergIndexV_sma3nz_amean_numeric | 0.85 |
| hammarbergIndexV_sma3nz_stddevNorm_numeric | 0.78 |
| slopeV0500_sma3nz_amean_numeric | 1.04 |
| slopeV0500_sma3nz_stddevNorm_numeric | 1.12 |
| slopeV5001500_sma3nz_amean_numeric | 2.40 |
| slopeV5001500_sma3nz_stddevNorm_numeric | 1.69 |
| spectralFluxV_sma3nz_amean_numeric | 0.92 |
| spectralFluxV_sma3nz_stddevNorm_numeric | 0.86 |
| mfcc1V_sma3nz_amean_numeric | 0.86 |
| mfcc1V_sma3nz_stddevNorm_numeric | 0.99 |
| mfcc2V_sma3nz_amean_numeric | 0.78 |
| mfcc2V_sma3nz_stddevNorm_numeric | 0.96 |
| mfcc3V_sma3nz_amean_numeric | 0.90 |
| mfcc3V_sma3nz_stddevNorm_numeric | 1.12 |
| mfcc4V_sma3nz_amean_numeric | 0.88 |
| mfcc4V_sma3nz_stddevNorm_numeric | 0.92 |
| alphaRatioUV_sma3nz_amean_numeric | 1.11 |
| hammarbergIndexUV_sma3nz_amean_numeric | 0.88 |
| slopeUV0500_sma3nz_amean_numeric | 1.05 |
| slopeUV5001500_sma3nz_amean_numeric | 7.99 |
| spectralFluxUV_sma3nz_amean_numeric | 1.10 |
| loudnessPeaksPerSec_numeric | 1.34 |
| VoicedSegmentsPerSec_numeric | 24.66 |
| MeanVoicedSegmentLengthSec_numeric | 1.23 |
| StddevVoicedSegmentLengthSec_numeric | 1.35 |
| MeanUnvoicedSegmentLength_numeric | 3.55 |
| StddevUnvoicedSegmentLength_numeric | 1.94 |
| equivalentSoundLevel_dBp_numeric | 1.17 |

**Supplementary Table 2. Gini-importance scores of the acoustic parameters in the psychotic symptoms classifier (positive vs negative symptoms**

| **Parameter** | **Gini-importance** |
| --- | --- |
| F0semitoneFrom27.5Hz_sma3nz_amean_numeric | 0.46 |
| F0semitoneFrom27.5Hz_sma3nz_stddevNorm_numeric | 0.42 |
| F0semitoneFrom27.5Hz_sma3nz_percentile20.0_numeric | 0.37 |
| F0semitoneFrom27.5Hz_sma3nz_percentile50.0_numeric | 0.58 |
| F0semitoneFrom27.5Hz_sma3nz_percentile80.0_numeric | 0.35 |
| F0semitoneFrom27.5Hz_sma3nz_pctlrange02_numeric | 0.42 |
| F0semitoneFrom27.5Hz_sma3nz_meanRisingSlope_numeric | 0.49 |
| F0semitoneFrom27.5Hz_sma3nz_stddevRisingSlope_numeric | 0.38 |
| F0semitoneFrom27.5Hz_sma3nz_meanFallingSlope_numeric | 0.31 |
| F0semitoneFrom27.5Hz_sma3nz_stddevFallingSlope_numeric | 0.32 |
| loudness_sma3_amean_numeric | 0.29 |
| loudness_sma3_stddevNorm_numeric | 0.56 |
| loudness_sma3_percentile20.0_numeric | 0.30 |
| loudness_sma3_percentile50.0_numeric | 0.53 |
| loudness_sma3_percentile80.0_numeric | 0.29 |
| loudness_sma3_pctlrange02_numeric | 0.25 |
| loudness_sma3_meanRisingSlope_numeric | 0.40 |
| loudness_sma3_stddevRisingSlope_numeric | 0.49 |
| loudness_sma3_meanFallingSlope_numeric | 0.40 |
| loudness_sma3_stddevFallingSlope_numeric | 0.34 |
| spectralFlux_sma3_amean_numeric | 0.21 |
| spectralFlux_sma3_stddevNorm_numeric | 0.28 |
| mfcc1_sma3_amean_numeric | 0.45 |
| mfcc1_sma3_stddevNorm_numeric | 0.42 |
| mfcc2_sma3_amean_numeric | 0.30 |
| mfcc2_sma3_stddevNorm_numeric | 0.37 |
| mfcc3_sma3_amean_numeric | 0.46 |
| mfcc3_sma3_stddevNorm_numeric | 1.12 |
| mfcc4_sma3_amean_numeric | 0.61 |
| mfcc4_sma3_stddevNorm_numeric | 0.27 |
| jitterLocal_sma3nz_amean_numeric | 0.52 |
| jitterLocal_sma3nz_stddevNorm_numeric | 0.97 |
| shimmerLocaldB_sma3nz_amean_numeric | 0.51 |
| shimmerLocaldB_sma3nz_stddevNorm_numeric | 0.55 |
| HNRdBACF_sma3nz_amean_numeric | 0.42 |
| HNRdBACF_sma3nz_stddevNorm_numeric | 0.44 |
| logRelF0H1H2_sma3nz_amean_numeric | 0.32 |
| logRelF0H1H2_sma3nz_stddevNorm_numeric | 0.42 |
| logRelF0H1A3_sma3nz_amean_numeric | 0.25 |
| logRelF0H1A3_sma3nz_stddevNorm_numeric | 0.39 |
| F1frequency_sma3nz_amean_numeric | 0.92 |
| F1frequency_sma3nz_stddevNorm_numeric | 0.73 |
| F1bandwidth_sma3nz_amean_numeric | 1.07 |
| F1bandwidth_sma3nz_stddevNorm_numeric | 0.28 |
| F1amplitudeLogRelF0_sma3nz_amean_numeric | 0.41 |
| F1amplitudeLogRelF0_sma3nz_stddevNorm_numeric | 0.64 |
| F2frequency_sma3nz_amean_numeric | 0.53 |
| F2frequency_sma3nz_stddevNorm_numeric | 0.85 |
| F2bandwidth_sma3nz_amean_numeric | 0.92 |
| F2bandwidth_sma3nz_stddevNorm_numeric | 0.48 |
| F2amplitudeLogRelF0_sma3nz_amean_numeric | 0.40 |
| F2amplitudeLogRelF0_sma3nz_stddevNorm_numeric | 0.68 |
| F3frequency_sma3nz_amean_numeric | 0.66 |
| F3frequency_sma3nz_stddevNorm_numeric | 1.01 |
| F3bandwidth_sma3nz_amean_numeric | 0.82 |
| F3bandwidth_sma3nz_stddevNorm_numeric | 0.41 |
| F3amplitudeLogRelF0_sma3nz_amean_numeric | 0.48 |
| F3amplitudeLogRelF0_sma3nz_stddevNorm_numeric | 0.70 |
| alphaRatioV_sma3nz_amean_numeric | 0.30 |
| alphaRatioV_sma3nz_stddevNorm_numeric | 0.91 |
| hammarbergIndexV_sma3nz_amean_numeric | 0.29 |
| hammarbergIndexV_sma3nz_stddevNorm_numeric | 0.52 |
| slopeV0500_sma3nz_amean_numeric | 0.49 |
| slopeV0500_sma3nz_stddevNorm_numeric | 0.50 |
| slopeV5001500_sma3nz_amean_numeric | 0.38 |
| slopeV5001500_sma3nz_stddevNorm_numeric | 0.54 |
| spectralFluxV_sma3nz_amean_numeric | 0.33 |
| spectralFluxV_sma3nz_stddevNorm_numeric | 0.38 |
| mfcc1V_sma3nz_amean_numeric | 0.40 |
| mfcc1V_sma3nz_stddevNorm_numeric | 0.76 |
| mfcc2V_sma3nz_amean_numeric | 0.52 |
| mfcc2V_sma3nz_stddevNorm_numeric | 0.59 |
| mfcc3V_sma3nz_amean_numeric | 0.41 |
| mfcc3V_sma3nz_stddevNorm_numeric | 0.66 |
| mfcc4V_sma3nz_amean_numeric | 0.27 |
| mfcc4V_sma3nz_stddevNorm_numeric | 0.36 |
| alphaRatioUV_sma3nz_amean_numeric | 0.82 |
| hammarbergIndexUV_sma3nz_amean_numeric | 0.47 |
| slopeUV0500_sma3nz_amean_numeric | 1.56 |
| slopeUV5001500_sma3nz_amean_numeric | 0.30 |
| spectralFluxUV_sma3nz_amean_numeric | 0.29 |
| loudnessPeaksPerSec_numeric | 0.28 |
| VoicedSegmentsPerSec_numeric | 0.57 |
| MeanVoicedSegmentLengthSec_numeric | 0.61 |
| StddevVoicedSegmentLengthSec_numeric | 0.48 |
| MeanUnvoicedSegmentLength_numeric | 0.27 |
| StddevUnvoicedSegmentLength_numeric | 0.20 |
| equivalentSoundLevel_dBp_numeric | 0.31 |
